# Altered Brain Volume, Microstructure Metrics and Functional Connectivity Features in Multiple System Atrophy

**DOI:** 10.3389/fnagi.2022.799251

**Published:** 2022-05-19

**Authors:** Yunxiang Ge, Weimin Zheng, Yujia Li, Weibei Dou, Shan Ren, Zhigang Chen, Zhiqun Wang

**Affiliations:** ^1^Department of Electronic Engineering, Beijing National Research Center for Information Science and Technology (BNRist), Tsinghua University, Beijing, China; ^2^Department of Radiology, Aerospace Center Hospital, Beijing, China; ^3^Department of Neurology, Dongfang Hospital, Beijing University of Chinese Medicine, Beijing, China

**Keywords:** multiple system atrophy, multimodal MRI, microstructure metrics, extended network-based statistics, support vector machine

## Abstract

In order to deeply understand the specific patterns of volume, microstructure, and functional changes in Multiple System Atrophy patients with cerebellar ataxia syndrome (MSA-c), we perform the current study by simultaneously applying structural (T1-weighted imaging), Diffusion tensor imaging (DTI), functional (BOLD fMRI) and extended Network-Based Statistics (extended-NBS) analysis. Twenty-nine MSA-c type patients and twenty-seven healthy controls (HCs) were involved in this study. First, we analyzed the whole brain changes of volume, microstructure, and functional connectivity (FC) in MSA-c patients. Then, we explored the correlations between significant multimodal MRI features and the total Unified Multiple System Atrophy Rating Scale (UMSARS) scores. Finally, we searched for sensitive imaging biomarkers for the diagnosis of MSA-c using support vector machine (SVM) classifier. Results showed significant grey matter atrophy in cerebellum and white matter microstructural abnormalities in cerebellum, left fusiform gyrus, right precentral gyrus and lingual gyrus. Extended-NBS analysis found two significant different connected components, featuring altered functional connectivity related to left and right cerebellar sub-regions, respectively. Moreover, the reduced fiber bundle counts at right Cerebellum_3 (Cbe3) and decreased fractional anisotropy (FA) values at bilateral Cbe9 were negatively associated with total UMSARS scores. Finally, the significant features at left Cbe9, Cbe1, and Cbe7b were found to be useful as sensitive biomarkers to differentiate MSA-c from HCs according to the SVM analysis. These findings advanced our understanding of the neural pathophysiological mechanisms of MSA from the perspective of multimodal neuroimaging.

## Introduction

Multiple system atrophy (MSA) is a progressive neurodegenerative disorder. Its pathological feature is the deposition of alpha synuclein-positive glial cytoplasmic inclusions (GCIs) in some specific regions, such as cerebellum, striatum and olivopontine structures (Brettschneider et al., 2017; Krismer et al., 2019). MSA patients might be associated with autonomic dysfunction. It is further divided into two sub-types, according to whether the patient shows cerebellar ataxia syndrome (MSA-c) or poor levodopa-responsive parkinsonian syndrome (MSA-p) (Gilman et al., 2008). Alpha synuclein-positive GCI has been shown to be associated with the development of MSA in recent studies. However, the pathological mechanism is not well understood, and it remains to be a big challenge to accurately diagnose MSA.

Magnetic resonance imaging (MRI) has been applied to reveal the structural patterns of MSA patients in previous researches (Matsusue et al., 2009; Deguchi et al., 2015; Krismer and Wenning, 2017; Chelban et al., 2019; Dash et al., 2019). As a result, atrophy of the cerebellum, pons, middle cerebellar peduncle (MCP) and putamen was found in MSA patients, reflecting the pathological changes of the disease (Matsusue et al., 2009; Deguchi et al., 2015). Furthermore, some MRI studies reported the “putaminal slit” sign which was associated with the accumulation of iron and gliosis and the “hot-cross bun” sign which was associated with the selective depletion of myelinated transverse pontocerebellar fibers and pontine neurons in different types of MSA patients (Krismer and Wenning, 2017; Chelban et al., 2019; Dash et al., 2019). In order to improve the diagnostic accuracy, more detailed grey matter abnormality has been found in research settings, among which the cerebellar volume atrophy is widely observed (Yang et al., 2019; Lin et al., 2020). However, some MSA patients might have no abnormal structure changes on MRI (Chelban et al., 2019). It is therefore necessary to search for more sensitive biomarkers from other MRI modalities to accurately diagnose the disease.

Diffusion tensor imaging (DTI) is a noninvasive neuroimaging technique that can provide quantitative information of the diffusion characteristics of the water molecules in the brain. Using DTI, white matter changes including a significant decrease in fractional anisotropy (FA) and increase in mean diffusivity (MD) was observed in corticospinal tracts, transverse pontocerebellar fibers, pons, putamen, middle cerebellar peduncles (MCP) and cerebellum in MSA patients (Loh et al., 2011; Rulseh et al., 2016; Dash et al., 2019; Beliveau et al., 2021).

Besides the volumetric and microstructural changes of MSA, resting state functional MRI (fMRI) has revealed abnormal functional activity and connectivity patterns related to MSA. Previous studies reported the disrupted default mode network (DMN), striatal-thalamo-cortical network, cerebello-thalamo-cortical network, sensorimotor network and visual associated network in MSA patients (You et al., 2011; Wang et al., 2017; Yao et al., 2017; Ren et al., 2019; Zheng et al., 2019). Furthermore, researchers detected altered network topology and graph theory attributes by resting state graph theoretical analysis (Morisi et al., 2018; Sako et al., 2019). Previous studies provided evidence for the hypothesis that MSA may be caused by the disconnection syndrome, indicating that the accumulated alpha-synuclein may destroy several specific networks, and result in the movement disorder and other clinical symptoms (Rosskopf et al., 2018).

Most of previous studies focused on structural changes of MSA. Only a few studies reported new functional findings, which might improve diagnostic accuracy and provide novel disease progression markers. However, structural or functional analysis alone cannot provide all the necessary information to accurately diagnose MSA. Thus, a multimodal approach combining these innovative techniques seems likely to offer the best biomarker for clinical diagnosis of MSA. We speculate that a combination of structural grey matter and white matter features, DTI microstructure metrics and functional connectivity calculated from resting-state fMRI might improve diagnostic accuracy of MSA. In order to explore the patterns of brain volume, microstructural and functional changes of MSA, we simultaneously applied structural (T1-weighted imaging), DTI, functional (BOLD fMRI) and extended Network-Based Statistics (extended-NBS) analysis in this study. First of all, we analyzed the whole brain changes of volume, microstructure, and functional connectivity in MSA-c patients. Then, the correlations between multimodal MRI features and clinical measurements were explored. Finally, classification is performed using support vector machine (SVM), in order to search for sensitive imaging biomarkers for the diagnosis of MSA.

## Materials and Methods

### Participants

The participant collection is similar as our previous study (Zheng et al., 2020). Thirty-four right-handed MSA-c patients and twenty-nine right-handed healthy controls (HCs) matched in age and gender were recruited. All the participants were from Dongfang Hospital of Beijing University of Chinese Medicine. The recruitment criteria for MSA-c patients in this study were based on the international diagnostic criteria, which was defined by American Academy of Neurology and American Autonomic Society (Gilman et al., 2008). The study included patients diagnosed as probable MSA and without hemorrhage, infarction, tumors, trauma, or severe white matter hyperintensity. Each patient was assessed by the Unified Multiple System Atrophy Rating Scale (UMSARS), Montreal Cognitive Assessment (MoCA), Hamilton Anxiety Scale (HAMA) and Hamilton Depression Scale (HAMD). The healthy control group was included according to the following criteria: (1) no neurological or psychiatric disorders; (2) no significant cognitive decline; (3) no visual, auditory or other neurological dysfunction; (4) had not received deep brain stimulation or surgical treatment; (5) MRI scan showed no motor impairment, vascular brain injury, brain tumor, and/or significant cortical and/or subcortical atrophy. In accordance with the Declaration of Helsinki, each of the subjects gave a written informed consent. The study was approved by the Medical Research Ethical Committee of Dongfang Hospital of Beijing University of Chinese Medicine.

Among the recruited thirty-four patients, one was later identified as mis-diagnosed (patient 23) and excluded. Patient 32 did not finish clinical assessment and was discarded. During MRI scanning, two patients (patient 26 and 34) and two controls (subject 25 and 28) did not complete structural scanning and were not included in further analysis. In correlation analysis, one more subject (patient 21) was identified as an outlier and was excluded. As a result, the experiment was conducted using data collected from twenty-nine MSA-c patients and twenty-seven healthy controls. The data exclusion procedure was visualized in Supplementary Figure 1.

### Data Acquisition

The image dataset was acquired using a GE 3.0T Discovery 750 scanner. 3D T1-weighted sequence parameters were as follows: repetition time (TR) = 8.2 ms, echo time (TE) = 3.17 ms, inversion time (TI) = 450 ms, flip angle (FA) = 12°, slices = 188, thickness = 1 mm, resolution = 256 × 256 matrix, voxel size = 1 mm × 1 mm× 1 mm. Diffusion tensor imaging was collected using the echo-planar imaging (EPI) sequence with 7 minutes scanning time. The imaging parameters were as follows: TR = 6,000 ms, TE = minimum, FA=12°, thickness = 3 mm, gap = 0 mm, FOV = 25.6 × 25.6 cm^2^, slices = 50, bandwidth = 250 kHz, resolution = 128 × 128 matrix. 64 diffusion-sensitive gradients in nonlinear directions were adopted, and the b values of the diffusion gradients were 0 s/mm^2^ and 1,000 s/mm^2^. The resting-state fMRI scanning lasted for 6 min with the following parameters: TR = 2,000 ms, TE = 30 ms, FA = 90°, FOV = 24 cm × 24 cm, resolution = 64 × 64 matrix, thickness = 3 mm, slices = 36, gap = 1 mm, voxel size = 3.75 mm × 3.75 mm × 3 mm, bandwidth = 2,232 Hz/pixel. During scanning, all participants were instructed to keep their eyes closed, move as little as possible, think of nothing in particular, and stay awake. In total, 6,480 functional images were acquired for each subject, corresponding to 180 volumes.

### Data Processing

**FIGURE 1:**
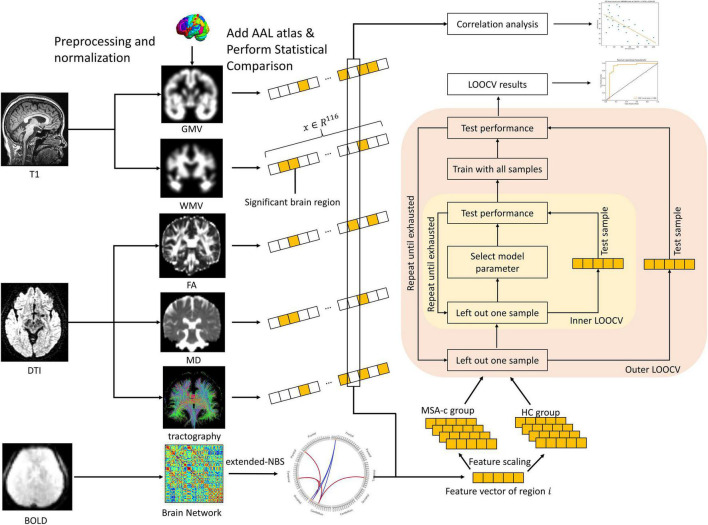
Method overview. Data from each modality was first preprocessed and normalized to the MNI152 standard space, producing a range of raw features. The AAL atlas was utilized to convert the raw features to feature vectors and statistical analysis was performed. We selected significant features for each brain region for further correlation as well as classification analysis. MNI, Montreal Neurological Institute; AAL, Automated Anatomical Labeling.

The method overview is shown in Figure 1. In order to merge multimodal features, data from each modality was first normalized to the standard Montreal Neurological Institute (MNI) 152 space with isotropic spatial resolution. We utilized the Automated Anatomical Labeling (AAL) atlas (Tzourio-Mazoyer et al., 2002) which parceled the brain and cerebellum into 116 areas for further analysis. For volumetric features extracted from T1 and DTI, values at voxels within each brain region volume were averaged as the feature associated with this brain area, and the whole brain feature was converted to a vector with 116 values, each corresponding to one brain region. For functional data, we constructed whole brain networks with 116 nodes by calculating functional connectivity between each pair of nodes. In the following subsections, we describe data processing methods for each modality.

#### T1-Weighted Imaging Analysis

T1-weighted images were preprocessed using Statistical Parametric Mapping 12 (SPM12)^1^ software in MATLAB 2018b (Math Works, Natick, MA, United States). First, the T1-weighted images were segmented into three components, including grey matter (GM), white matter (WM) and cerebrospinal fluid (CSF). Second, we utilized the Diffeomorphic Anatomical Registration through Exponentiated Lie algebra (DARTEL) algorithm to normalize images into the standard MNI 152 space with 1mm isotropic resolution. Finally, the normalized GM and WM components were modulated for nonlinear change and smoothed using a Gaussian kernel of 8 mm full width at half-maximum (FWHM).

We utilized GM and WM components for further analysis and applied the AAL atlas to convert the raw data to feature space. The weighted grey matter volume (GMV) and weighted white matter volume (WMV) for each brain region was extracted by averaging values at each voxel within each region. Since the grey matter and white matter components produced by SPM segmentation are probability maps, the average of values at voxels within each region can be viewed as a weighted volume. As a result, the whole-brain GMV and WMV of each subject was represented by a feature vector.

#### Diffusion Tensor Imaging Analysis

The preprocessing of DTI data was based on FSL (Smith et al., 2004). First, brain extraction was performed by bet2 and fslmaths. Then we corrected the eddy currents and movements in diffusion data. Finally, the DTI data was normalized to a 2mm-isotropic T1 template in the MNI 152 standard space.

We used an in-house software, Multi-Modal Data Processing System (MMDPS) (Wang, 2018) and the Dipy package (Garyfallidis et al., 2014) for DTI data processing. The Constrained Spherical Deconvolution (CSD) model (Tournier et al., 2007) was firstly fitted with the preprocessed DTI data. Then the probabilistic tracking was performed by following the possible trajectory pathway of neural fibers. Based on the tracking results, we applied the AAL atlas (Tzourio-Mazoyer et al., 2002) and calculated the number of fibers going through each brain region. The tractography feature was represented by a feature vector of number of fiber bundles crossing each region (fiber bundle counts). Besides, we also calculated the fractional anisotropy (FA) and mean diffusivity (MD) with Dipy, and converted them into feature vectors using the AAL atlas by averaging values at voxels within each region.

#### Resting-State fMRI Analysis

The preprocessing of fMRI data was similar as in our previous research (Zheng et al., 2020). We used DPARSFA (V4.3) (Yan and Zang, 2010) and SPM12 (V6906) to preprocess data. Firstly, we discarded the first 10 time points and performed slice timing correction. Then, head motion was corrected and the data was normalized to the BOLD EPI template in the standard MNI 152 space with 2mm isotropic resolution. The image was resampled to 3-mm isotropic voxels and spatially smoothed by a 4mm full-width half-maximum (FWHM) Gaussian kernel. We further removed the linear trend and nuisance covariates, including head motions, cerebral fluid, white matter and the global signal. Finally, the signal time series were filtered and signals within 0.01-0.08 Hz were kept.

We used an in-house software, Multi-Modal Data Processing System (MMDPS) (Wang, 2018) for further data processing. The brain network nodes were defined using the AAL atlas (Tzourio-Mazoyer et al., 2002) with 116 areas. The BOLD time series was averaged within each brain region and functional connectivity was calculated as the Pearson correlation coefficient between each pair of regions. The functional connectivity is defined as the edge in the network. As a result, we built a brain network with 116 nodes for each subject.

### Statistical Analysis

Two-sample t-tests were used to find significant different brain regions for grey matter volume (GMV), white matter volume (WMV), FA, MD as well as fiber bundle count results. The feature values averaged at each region volume were extracted from the two groups and group comparison was performed. All regions across the whole brain were tested and the Benjamini - Hochberg procedure was adopted to control the False Discovery Rate (FDR). The feature at a specific region was declared significant if the p-value is less than 0.001 (FDR corrected). Significant features were identified for metrics extracted from each MRI modality.

In order to evaluate clinical relativity of imaging features, we calculated Pearson correlation coefficients between significant features (*p* < 0.001, FDR corrected) and UMSARS-total scores of MSA-c patients. Features whose correlation p-value < 0.001 were selected for further discussion. During correlation analysis, we also inspected the distribution of significant features and clinical measurements by drawing the quantile-quantile plot (Q-Q plot) in order to examine for possible outliers in correlation. The statistical analysis, including two-sample t-tests between groups and correlation between features and clinical variables, was performed using the SciPy module, while multiple comparison correction was performed using the statsmodels module.

For functional network analysis, we utilized the extended network-based statistics (extended-NBS) (Zalesky et al., 2010; Ge et al., 2021) to evaluate functional connectivity changes in MSA patients. Firstly, the same connection was selected from the two groups and two-sample t-test was performed. This comparison was repeated for each connection in the network. As a result, each connection was associated with a t-statistic and *p-value*. A difference network whose edges were *p*-values obtained from the two-sample t-test was constructed. Then, connections whose *p*-value is larger than or less than a pre-defined *p*-value threshold were set to zero or one, respectively, producing a binary difference network. Connected components (CC) in the binary difference network were identified as a set of inter-connected regions. In order to decide whether a CC is significant, permutation test was performed. During each permutation, group label was randomly shuffled and the same procedures were performed, including comparing each connection, constructing binary difference networks, identifying CCs and storing the maximal size of CCs. The size of a connected component is defined as the number of regions within the CC. The permutation was performed 5,000 times, yielding an empirical distribution of the CC size. The empirical p-value of the original CC was calculated as the ratio of the number of permutations whose maximal CC size was larger than the original CC size, against total permutation number. Formally, let *CC*_*size*_*i*_ denote the maximal CC size at the i-th permutation, *CC*_*size*_0_ denote the original CC size. The empirical *p*-value is calculated as


$$E\,m\,p\,\_\,p\,v\,a\,l=\frac{\sum i\,n\,d\,\left(C\,C\,\_\,s\,i\,z\,e_{i}\right)}{5000}$$

Where


$$ind\left(CC\_size_{i}\right)=\{\begin{array}{cc} 1, & if\;CC\_size_{i}>CC\_size_{0}\\ 0, & otherwise \end{array}$$

If the empirical *p-value* was less than 0.05, the original CC was declared significant. During experiment, we varied the pre-defined *p*-value threshold from 0.0001 to 0.001 with a step size of 0.0001. Results obtained at *p*-value threshold 0.0001 were taken for further discussion.

### Classification

We constructed classifiers based on the significant features of each brain region in order to locate regions with biomarker potentials. The significant results produced by statistical analysis were taken as features for classification. In order to incorporate as many features as possible, we used less strict significance criteria compared to statistical analysis. Specifically, each region was associated with a list of significant features obtained from two-sample t-tests of features of each modality (*p* < 0.05, FDR corrected). The functional connections related to this region was also included if this region is involved in the significant CC when *p*-value threshold equals 0.001. For example, if region A is significant different in GMV, FA, and MD, then the feature vector of region A would contain [GMV (A), FA (A), MD (A)]. Moreover, if region A lies within the significant CC, the feature vector would further contain functional connections in the significant CC that directly related to region A. Before classification, the feature was standardized by removing the mean and scaling to unit variance.


$$\hat{x}=\frac{x-\mu }{s}$$

Where *x* stands for the feature vector, μ stands for the mean and *s* stands for the standard deviation.

We utilized the linear Support Vector Machine (SVM) as classifier and performed nested leave-one-out cross validation (LOOCV) for parameter selection and testing. In the outer LOOCV, one sample was left out as test sample and other samples were fed into the inner LOOCV, where another sample was selected as validation sample. The data left was used to train a classifier with different parameters. The model parameter, C, was varied in range [0.001, 0.01, 0.1, 1, 10, 100, 500, 1000, 5000, 10000]. After selecting the best parameter using the validation sample, all samples in the inner LOOCV were utilized to train the SVM with the selected parameter and the model was tested by the left-out sample in the outer LOOCV. The leave-one-out procedure was repeated until all samples have been left out once. We calculated accuracy, sensitivity and specificity for model comparison. Their definitions are as follows.


$$A\,c\,c\,u\,r\,a\,c\,y=\frac{T\,P+T\,N}{T\,P+T\,N+F\,P+F\,N}$$

$$S\,e\,n\,s\,i\,t\,i\,v\,i\,t\,y=\frac{T\,P}{T\,P+F\,N}$$

$$S\,p\,e\,c\,i\,f\,i\,c\,i\,t\,y=\frac{T\,N}{T\,N+F\,P}$$

Where TP stands for true positive, TN stands for true negative, FP stands for false positive and FN stands for false negative.

We also plotted the Receiver Operating Characteristic (ROC) curve and calculated the area under the curve (AUC) value for the classifier trained at each region. The training procedure was repeated for each brain region and the results were sorted by classification accuracy.

## Results

### Demographics

Twenty-nine MSA-c patients and twenty-seven healthy controls were involved in the current study. Table 1 showed the demographic and clinical information for these subjects. There was no significant difference in age and gender between MSA-c patients and healthy controls (*p* > 0.05).

**TABLE 1:** Clinical and demographical data.

	MSA-c (*n* = 29)	Control (*n* = 27)	*p*-value
Age, years	57.62*±6.00^#^	57.37*±5.64^#^	0.875^a^
Gender, male/female	18/11	11/16	0.110^b^
UMSARS-I	17.34*±5.65^#^	NA^e^	
UMSARS-II	17.07*±5.66^#^	NA	
UMSARS- total	34.41*±10.42^#^	NA	
Disease duration, years	2.34*±1.49^#^	NA	
MoCA	24.17*±3.76^#^	NA	
HAMA	13.33*±6.43^#^	NA	
HAMD	13.94*±8.61^#^	NA	
Genetic history of MSA	28Y^c^/1N^d^	NA	
Vertical gaze palsy	9Y/20N	NA	

*UMSARS, Unified Multiple System Atrophy Rating Scale; MoCA, Montreal Cognitive Assessment; HAMA, Hamilton Anxiety Scale; HAMD, Hamilton Depression Scale; MSA, Multiple System Atrophy.*

*represents average value;

*^#^stands for standard deviation;*

*^a^represents Independent Samples Test;*

*^b^represents Chi-Square Tests;*

*^c^represents Yes*,

*^d^represents No;*

*^e^represents Not Applicable.*

### Feature Differences and Correlations

**FIGURE 2:**
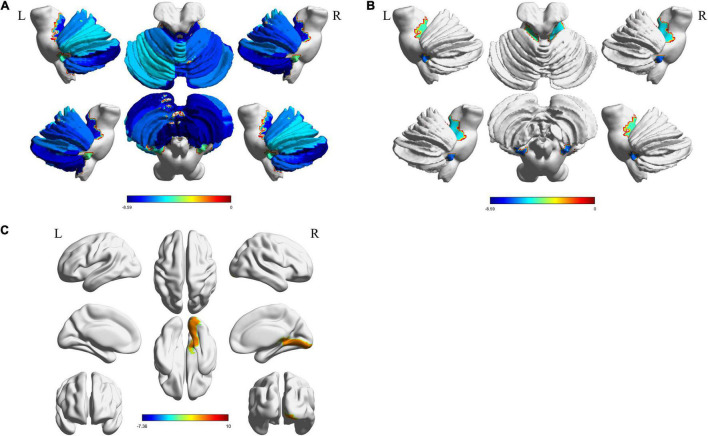
Significant regions found from T1. The t-statistics of each significant region were visualized for each feature. **(A)** Decreased GMV in cerebellum; **(B)** Decreased WMV in cerebellum; **(C)** Increased WMV in cerebrum area. GMV, gray matter volume; WMV, white matter volume.

**FIGURE 3:**
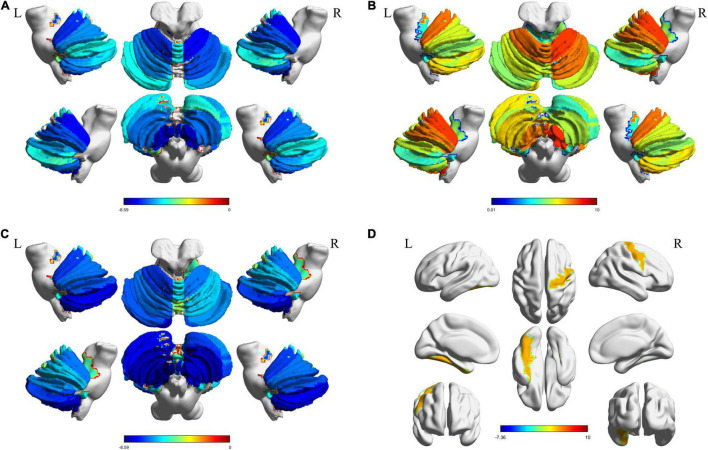
Significant regions found from DTI. The t-statistics of each significant region were visualized for each feature. **(A)** Decreased FA in cerebellum; **(B)** Increased MD in cerebellum; **(C)** Reduced fiber bundle counts in cerebellum; **(D)** Increased MD in cerebrum areas. DTI, Diffusion tensor imaging; FA, fractional anisotropy; MD, mean diffusivity.

Structural atrophy and white matter microstructural alterations in cerebellar and cerebral regions was identified in MSA-c patients. GMV at all subregions of the cerebellum were significantly decreased (*p* < 0.001, FDR corrected) in MSA-c patients (Figure 2A). WMV obtained from T1 showed significant decrease at bilateral Cerebellum_3 (Cbe3) and bilateral Cerebellum_10 (Cbe10) (Figure 2B). The right lingual gyrus, however, showed increased WMV (Figure 2C). On the other hand, features extracted from DTI reflected white matter alteration related to MSA-c patients. We identified reduced FA, fiber bundle count and increased MD at a range of cerebellar regions (Figures 3A–C). Increased MD in the left fusiform (FFG) and right precentral gyrus (PreCG) was also found in MSA-c patients (Figure 3D). The detailed statistics as well as significant region lists for each feature were reported in Supplementary Tables 1–5.

**FIGURE 4:**
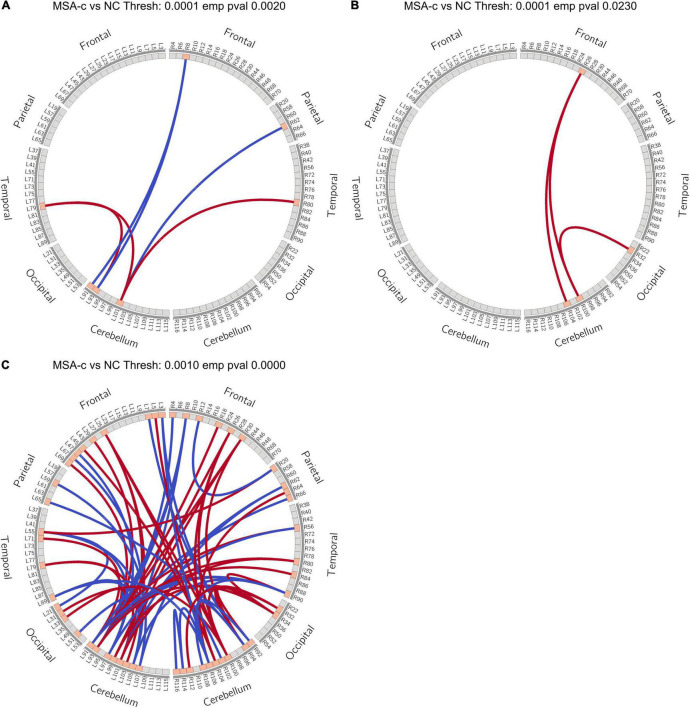
Extended-NBS results. **(A,B)** Significant different connected components obtained at *p*-value threshold of 0.0001 with empirical *p*-value of 0.0020 and 0.0230, respectively; **(C)** Significant different connected component obtained at *p-value* threshold of 0.0010 with empirical *p*-value less than 0.0001. Red and blue lines stand for increased and decreased connectivity, respectively. Extended-NBS, Extended Network-Based Statistics.

We constructed functional brain networks and compared between the two groups using extended-NBS. When varying the p-value threshold from 0.0001 to 0.001, several significant connected components whose empirical *p*-value was less than 0.05 were identified. The complete results were shown in Supplementary Figure 2. The first component contained increased connectivity alone, mainly consisting connections related to the right cerebellum. The second component featured connections related to the left cerebellum, and contained both increased and decreased connectivity. Another component that appeared at *p*-value threshold of 0.0002 contained decreased connections related to the right cerebellum, and was further merged into the second component as the *p*-value threshold increased to 0.0004. The first component was also incorporated into the second component when the *p*-value threshold increased over 0.0009. Component at *p*-value threshold 0.0010 was used as features for classification, which contained information provided in all three significant different components (Figure 4C).

When *p*-value threshold equals 0.0001, two significant different connected components were identified. The first component contained both increased and decreased connections related to sub-regions of the left cerebellum (Figure 4A). Decreased connections include FC between right middle frontal gyrus (MFG) and left Cerebellum_Crus1 (Cbe1), Cerebellum_2 (Cbe2), and FC between right inferior parietal lobe (IPL) and left Cerebellum_7b (Cbe7b). Increased connections are FC between left Cbe7b and bilateral heschl gyrus (HES), and FC between left Cbe2 and left HES. The second component contained increased connections related to sub-regions of the right cerebellum (Figure 4B). The increased connections are FC between right medial orbitofrontal cortex (mOFC) and right Cerebellum_6 (Cbe6), Cerebellum_8 (Cbe8), and FC between right anterior cingulate cortex (ACC) and right Cbe6.

**FIGURE 5:**
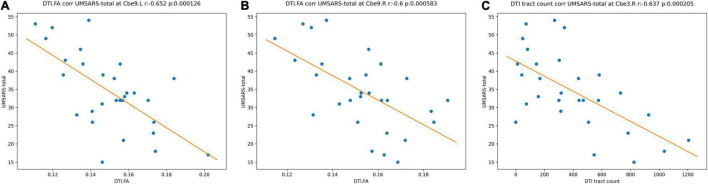
Correlations between the MRI significant features and UMSARS-total scores in MSA-c patients. **(A)** FA values of left Cbe9; **(B)** FA values of right Cbe9; **(C)** fiber bundle counts of right Cbe3. MRI, magnetic resonance imaging; UMSARS, Unified Multiple System Atrophy Rating Scale; MSA-c, cerebellum-type of multiple system atrophy; Cbe, Cerebellum; FA, fractional anisotropy.

Among the identified significant features, we further performed correlation analysis with UMSARS-total scores. Patient 21 was identified as an outlier by using Q-Q plot, which was excluded from all statistical and classification analysis. The outlier identification procedures were reported in the Supplementary Material. After excluding the outlier, we identified that the fiber bundle count of right Cerebellum_3 (Cbe3) and FA values at bilateral Cerebellum_9 (Cbe9) had negative significant correlation with clinical measurements (*p* < 0.001, Figure 5).

### Classification Results

**FIGURE 6:**
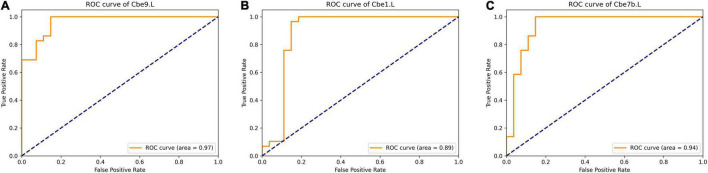
ROC curve of selected regions when classifying with significant features of each region. **(A)** left Cerebellum_9; **(B)** left Cerebellum_Crus1; **(C)** left Cerebellum_7b. ROC, Receiver Operating Characteristic.

For each brain region, we selected significant different features (*p* < 0.05, FDR corrected) from each modality, and combined functional connectivity related to this region from extended-NBS result at *p*-value threshold of 0.0010, forming the feature vector for classification. The linear support vector machine with embedded leave-one-out cross validation was performed to evaluate the potential value of diagnosis. The SVM analysis was repeated for each region and results were summarized in Supplementary Table 6. Here we report regions whose classification accuracy is higher than 0.90. By using significant features from all modalities, we found left Cbe9 reached the highest classification accuracy (0.928), with 1.000 sensitivity and 0.852 specificity. We also identified left Cerebellum_Crus1 (Cbe1) and left Cbe7b with 0.911 accuracy when classifying patients without white matter volume features. The classification results of these regions as well as the modalities involved were shown in Table 2. We also plotted the receiver operating characteristics (ROC) curves of these regions in Figure 6.

**TABLE 2:** Classification results of selected regions. The features were significant results associated with each region.

Regions	Features	AUC	Accuracy	Sensitivity	Specificity
Cbe9.L	GMV, WMV, FA, MD, fiber bundle counts, FC	0.97	0.928	1.000	0.852
Cbe1.L	GMV, FA, MD, fiber bundle counts, FC	0.89	0.911	1.000	0.815
Cbe7b.L	GMV, FA, MD, fiber bundle counts, FC	0.94	0.911	0.966	0.852

*Cbe, Cerebellum; GMV, grey matter volume; WMV, white matter volume; FA, fractional anisotropy; MD, mean diffusivity; FC, functional connectivity; AUC, area under the curve.*

## Discussion

### Major Findings

By simultaneously applying structural (T1-weighted imaging), DTI, functional (BOLD fMRI) and extended-NBS analysis, we found significant grey matter atrophy in cerebellum and white matter microstructural abnormalities in cerebellum, left FFG and right PreCG and lingual gyrus. Extended-NBS analysis revealed two significant connected components, featuring altered functional connectivity related to left and right cerebellar sub-regions, respectively. Moreover, the reduced fiber bundle counts of right Cbe3 and decreased FA values of bilateral Cbe9 were negatively associated with UMSARS-total scores. Finally, we found that the significant features of left Cbe9, Cbe1 and Cbe7b could be used as sensitive biomarkers to differentiate MSA-c from HCs according to the SVM analysis. The results of this study may help us to understand the neural pathophysiological mechanism of MSA from the perspective of multimodal neuroimaging.

### Structural Atrophy, White Matter Microstructural and Functional Abnormalities in MSA-c Patients in the Resting State

Significant grey matter atrophy and white matter microstructural abnormalities were found in most of the cerebellum subregions in MSA-c patients in the current study. It was shown that glial cytoplasmic inclusion body associated oligodendrocyte disease is the main pathological process of MSA (Wenning et al., 2008), and the definitive histopathological manifestation of MSA-c is mainly found in the cerebellum. In a recent study, the researchers found grey matter atrophy and white matter degeneration of cerebellum in MSA-c patients, which was consistent with the current study (Dash et al., 2019). It has been reported that the cerebellum plays an important role in the sensorimotor circuit (Timmann and Daum, 2010). Morphological changes such as structural atrophy may be caused by the primary disease process induced by pathological deposition. Additionally, increased MD in left FFG and right PreCG were found in this study. As a part of the visual association cortex, the fusiform gyrus is mainly responsible for visual association, episodic memory consolidation and visual image processing. It also participates in the regional network supporting oral declarative memory (Bremner et al., 2004; Malhi et al., 2007; Fusar-Poli et al., 2009; Tao et al., 2013; Kukolja et al., 2016). Some previous studies have shown that visual information is involved in regulating sensory and motor function (Anguera et al., 2011; Seidler and Carson, 2017; Fisher et al., 2019). Functional changes in visual-related areas have also been reported in one of our previous studies (Zheng et al., 2019). The anatomical changes in the visual-related region implied that the visual association cortex may be involved in regulating motor function following MSA, although the exact mechanism remains unclear.

Some early studies reported that the cerebellum’s main function was motor control (Andermann et al., 1975; Takagi et al., 1998), but recently many researchers reported that different subregions of cerebellum are involved in the formation of different functions, such as cognition, behavior and learning (Strick et al., 2009; Reeber et al., 2013). Consistent with previous studies, functional changes in cognitive-related cerebellar subregions (Cbe1, Cbe7b and Cbe8) were observed in current study (Desmond et al., 1997; Chen and Desmond, 2005a,b; Kirschen et al., 2005; Hayter et al., 2007). MFG and IPL as the primary hubs of the DMN (Buckner et al., 2008; Ward et al., 2014), are responsible for cognitive functions. The decreased FC between right MFG and left Cbe1, Cbe2, as well as between right IPL and left Cbe7b in the present study may suggest the cognitive dysfunction in MSA-c patients, which is partly consistent with one of our previous studies (Zheng et al., 2019). In a previous study, researchers found reduced activity in the mid-prefrontal cortex and altered functional connections to the insula, precuneus and inferior parietal in MSA patients (Yang et al., 2020). By using independent component analysis (ICA) and dual-regression analysis, researchers found the cognitive deficits may be caused by reduced cerebello-prefrontal and cerebello-amygdala functional connections in MSA (Kawabata et al., 2019). Our findings provide further evidence of cognitive impairment in MSA-c patients.

On the other hand, increased functional connectivity between left Cbe7b and bilateral HES, as well as between right mOFC and right Cbe6, Cbe8, and between right ACC and right Cbe6 were also found in the present study. Previous researches show that the ACC plays an important role in the processing of emotions (Albert et al., 2012) and is linked to mood disorders, including anxiety and depression associated with chronic pain (Barthas et al., 2015). In this study, more than 90% of the MSA patients had anxiety and depression, which may be the reason for the increased FC related to ACC. Additionally, studies have shown that individuals with non-suicidal self-injury behavior show hyperactivation in frontal areas, including the mOFC (Osuch et al., 2014). This indicates that psychological changes of MSA patients might cause functional alterations. In addition, HES belongs to the auditory cortex in humans. Studies have reported that MSA patients have a tendency to increase high-frequency auditory thresholds (Scarpa et al., 2020) and low-frequency auditory thresholds (Omichi et al., 2016; Scarpa et al., 2020), although it has also been reported that MSA patients do not experience significant auditory changes (Ikeda et al., 2013). As participants in this study did not receive audio-vestibular examination, we can only speculate that the increased functional connectivity is due to functional compensation. Further researches on auditory and vision function in MSA are needed.

### Relationships Between the Multimodal Magnetic Resonance Features and Clinical Performances

In current study, negative associations were found between clinical measurements (UMSARS-total scores) and the reduced fiber bundle counts of right Cbe3 and decreased FA values of bilateral Cbe9, suggesting clinical relevance of white matter microstructural abnormalities in MSA-c. The white matter microstructural abnormalities in these regions might be used as significant imaging markers for assessing the movement disorders.

### The Biomarkers to Differentiate MSA-c Patients From Healthy Controls Using Support Vector Machine Analysis

In current study, we first simultaneous applied the structural, DTI and extended-NBS analysis on MSA-c patients and HCs, and then used all significant features of each of the 116 regions to classify the two groups. Methods such as multilayer graph analysis and fusion ICA have been proposed to merge multimodal MRI data. However, multilayer graph analysis mainly works for merging brain networks constructed from different modalities (Battiston et al., 2017; Mandke et al., 2018). And fusion ICA is useful in combining contrast maps obtained from task fMRI data (Calhoun et al., 2006; Sui et al., 2013). In this work, we collected resting-state fMRI and constructed brain networks to model functional activities. Moreover, the features used in this work are not limited to networks. We consider regional features extracted from T1-weighted and DTI data, which lacks pairwise interaction information between regions. As a result, the AAL atlas was utilized to convert features from different modalities to the atlas space or the feature space. Regional features and networks were represented as feature vectors and matrices. And significant features identified from statistical analysis were used for classification training.

Results showed that the significant features of left Cbe9, Cbe1 and Cbe7b could distinguish MSA-c from HC with relative high accuracy. As previous studies reported, Cbe9 is thought to be crucial for visual guidance of movement (Glickstein et al., 1994). The function of Cbe1 is predominantly sensorimotor, whereas Cbe7b contributes to higher-level processes (Schmahmann, 1991, 1996, 2004; Schmahmann et al., 2009). Current result has an important implication since these regions could be used as valuable imaging biomarkers for the early diagnosis and prognosis assessment of MSA-c patients.

### Future Considerations

This work has several limitations. First, the present study showed alterations in visual, auditory and cognitive related regions in MSA patients. However, in this study, we only focused on changes in motor function. A study including more subjects with visual and cognitive function evaluations will be conducted in the future. Next, we have only incorporated one subtype of MSA in this study. In the future, exploring MSA of different subtypes (parkinsonian and cerebellar variants) would be helpful to deepen the understanding of pathological mechanism of MSA. Moreover, a longitudinal design including the pre-symptomatic patients is of great significance to elucidate the progression of MSA. Also, it would be more clinically meaningful to consider a dynamic and longitudinal context in future studies, including the neuroplasticity mechanisms in MSA and other neurodegenerative diseases (NDDs) (Gatto et al., 2019, 2021; Gatto, 2020). We also note that novel multimodal fusion methods that combine regional features and brain networks at the same time could possibly further help to improve classification accuracy.

## Conclusion

In conclusion, by simultaneously applying structural (T1-weighted imaging), DTI, functional (BOLD fMRI) and extended-NBS analysis to differentiate MSA-c from HCs, we observed significant grey matter atrophy in cerebellum and white matter microstructural abnormalities in cerebellum and several cerebrum regions, as well as altered functional connectivity between several cerebellum subregions and cerebrum regions in MSA-c patients. In addition, significant negative correlations were found between the UMSARS scores and white matter microstructural abnormalities in several cerebellum subregions of MSA-c patients. Finally, we found the significant features of left Cbe9, Cbe1 and Cbe7b could be used as useful imaging biomarkers to distinguish MSA-c from HCs according to the SVM analysis. The results of this study added new evidence for the structural atrophy, white matter microstructural and functional abnormalities of MSA, which may help us to understand the neural pathophysiological mechanism and provide potential biomarkers and new ideas for the accurate diagnosis and treatment of MSA in the future.

## Data Availability Statement

The raw data analyzed in this manuscript are not publicly available due to local regulations and ethical concerns. Requests to access the processed features should be directed to ZW, wangzhiqun@126.com and WD, douwb@tsinghua.edu.cn.

## Ethics Statement

The studies involving human participants were reviewed and approved by the Medical Research Ethical Committee of Dongfang Hospital of Beijing University of Chinese Medicine. The patients/participants provided their written informed consent to participate in this study.

## Author Contributions

ZW, WD, and ZC were responsible for the study concept and design. SR contributed to the acquisition of MRI data. YG devised the method framework, conducted data analysis, and assisted with data interpretation. WZ assisted with T1 data analysis and was responsible for data interpretation. YL assisted with DTI data analysis. WZ, YG, WD, and ZW drafted the manuscript. All authors provided critical revision of the manuscript for important intellectual content, critically reviewed content and approved final version for publication.

## Conflict of Interest

The authors declare that the research was conducted in the absence of any commercial or financial relationships that could be construed as a potential conflict of interest.

## Publisher’s Note

All claims expressed in this article are solely those of the authors and do not necessarily represent those of their affiliated organizations, or those of the publisher, the editors and the reviewers. Any product that may be evaluated in this article, or claim that may be made by its manufacturer, is not guaranteed or endorsed by the publisher.

## Footnotes

1
http://www.fil.ion.ucl.ac.uk/spm/
