# Supplementary material for: Altered Brain Volume, Microstructure Metrics and Functional Connectivity Features in Multiple System Atrophy
Source: Front Aging Neurosci. 2022 May 19;14:799251. doi: 10.3389/fnagi.2022.799251 (PMC9162384; doi:10.3389/fnagi.2022.799251)
Supplement: Supplementary file 1 [file Data_Sheet_1.DOCX]

# Supplementary Material

##
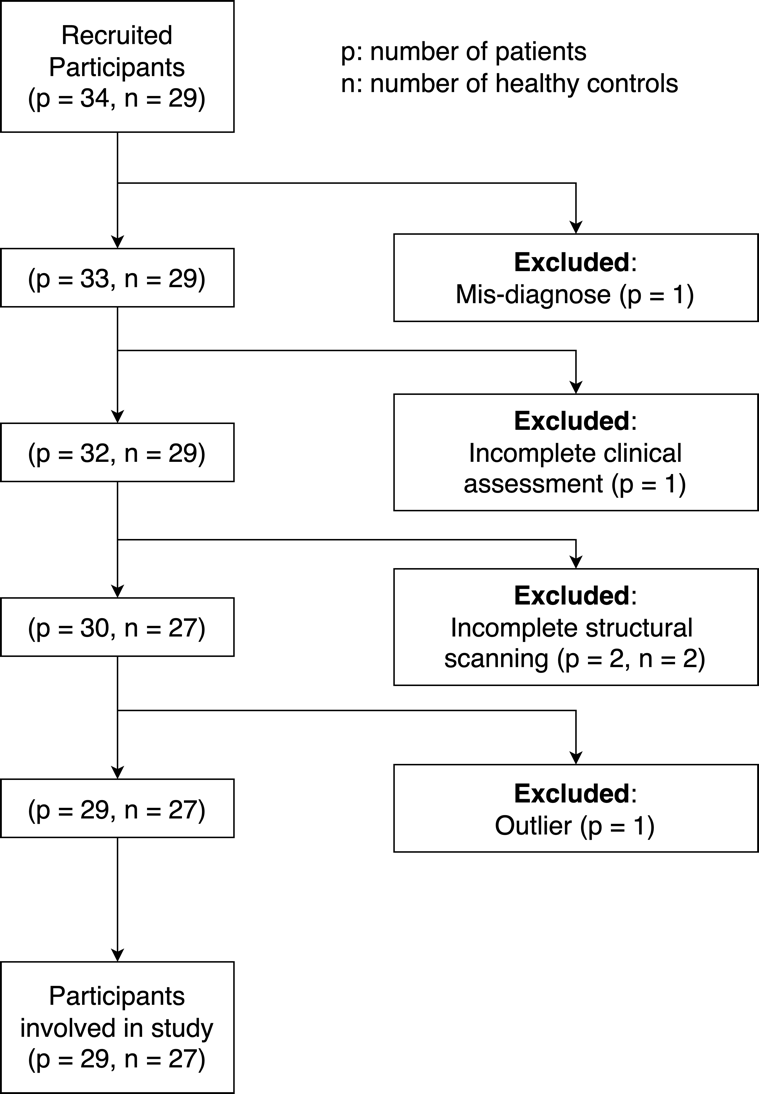
Supplementary Figures

**Supplementary Figure 1**. The subject exclusion procedure. Initially thirty-four MSA-c patients and twenty-nine healthy controls were enrolled. Patient 23 was later identified as mis-diagnosed. Patient 32 did not finish clinical assessment. Patient 26, 34 and subject 25, 28 did not finish structural scanning. Patient 21 was identified as an outlier during correlation analysis. As a result, twenty-nine MSA-c patients and twenty-seven healthy controls remained in the study.

**Supplementary Figure 2**. Extended Network-Based Statistics Result. Significant different connected components under different p-value thresholds were visualized. From left to right each column represents results obtained under p-value threshold (Thresh) from 0.0001 to 0.001. Each row stands for a specific component. Empty circle means no significant result (NS). Red and blue lines stand for increased and decreased connectivity, respectively. The empirical p-value (emp pval) was shown in the title of each sub-plots.

The figure was uploaded as a separate file (filename: SM Figure 1 - NBS results CMSA 29 vs NC 27.png).

## Outlier identification

In this study, we recruited thirty-four MSA-c patients and twenty-nine healthy controls (HC). Two patients were excluded due to incomplete clinical assessment and mis-diagnosis. During data collection, another two patients and two healthy controls were discarded due to incomplete structural scanning. As a result, thirty MSA-c patients and twenty-seven healthy controls remained.

We performed correlation analysis by calculating correlation coefficients between significant features of patients and UMSARS-total scores. The significant features were identified using two-sample t-test with FDR correction. Features whose p-value < 0.001 were selected. Seven features were found to show significant correlation with clinical measurements. However, we identified an obvious outlier and confirmed it with quantile-quantile plot (Q-Q plot). The correlation results, along with their Q-Q plots, were shown here (Supplementary Figure 3 - 9).


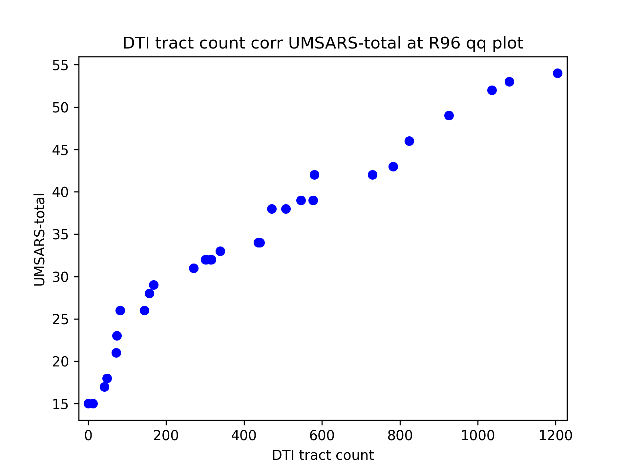

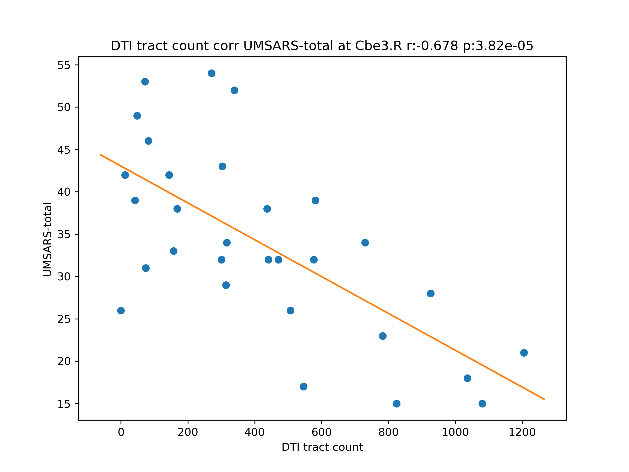


**Supplementary Figure 3**. Correlation result (left) and Q-Q plot (right) of DTI tract count at R96 (Cbe3.R). No obvious outlier was found.


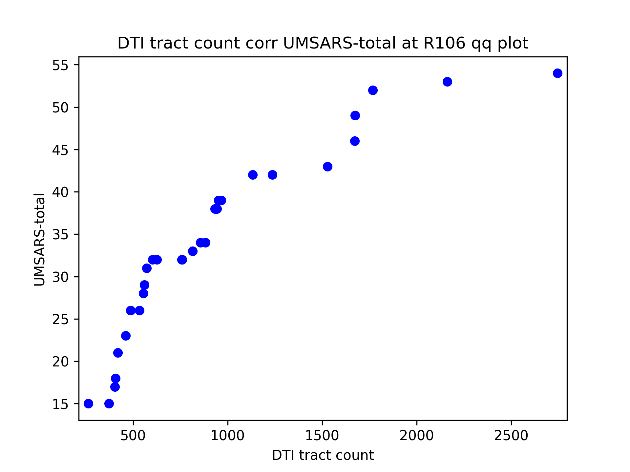

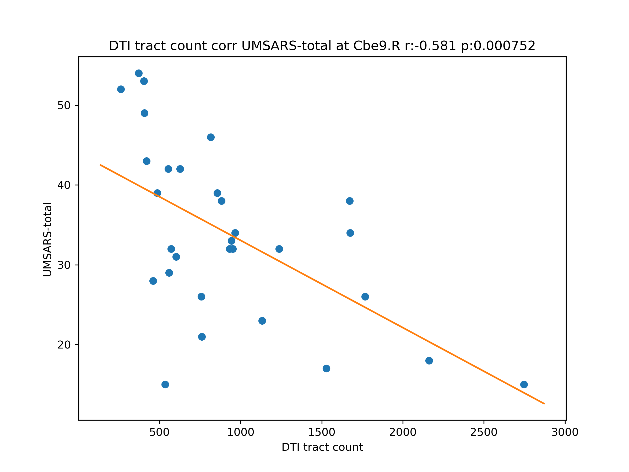


**Supplementary Figure 4**. Correlation result (left) and Q-Q plot (right) of DTI tract count at R106 (Cbe9.R). One obvious outlier (patient 21) was found.


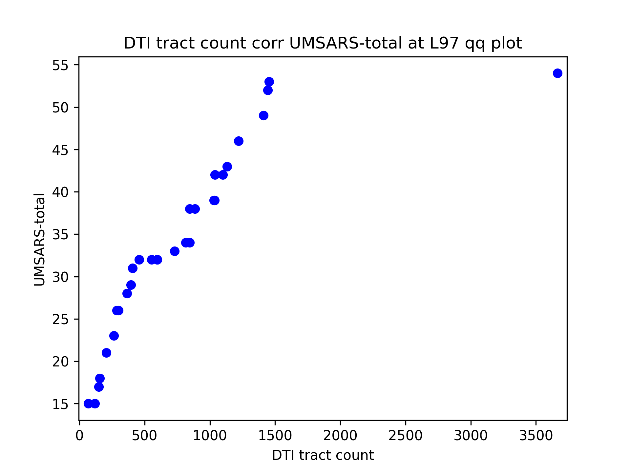

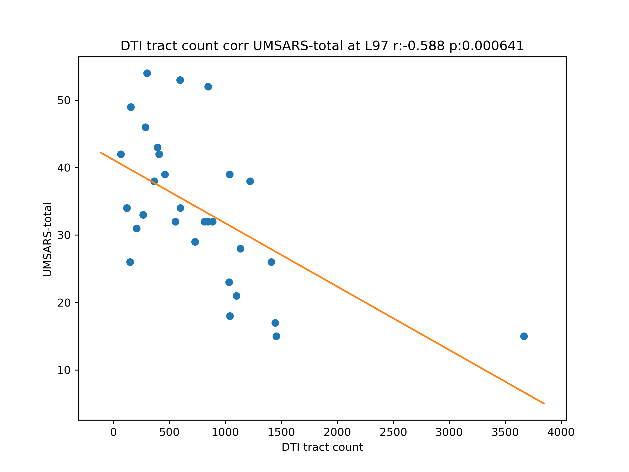


**Supplementary Figure 5**. Correlation result (left) and Q-Q plot (right) of DTI tract count at L97 (Cbe4_5.L). One obvious outlier (patient 21) was found.


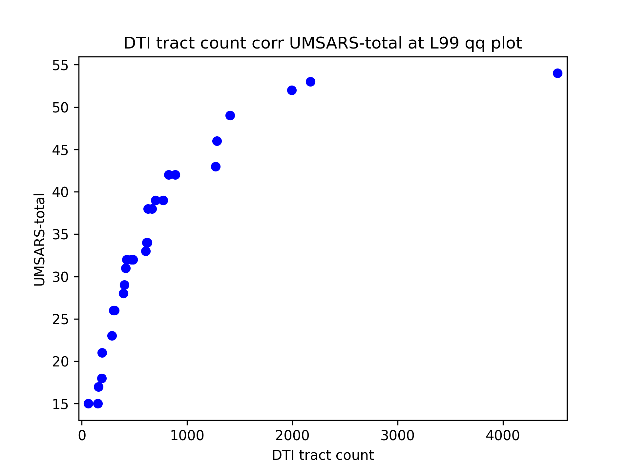

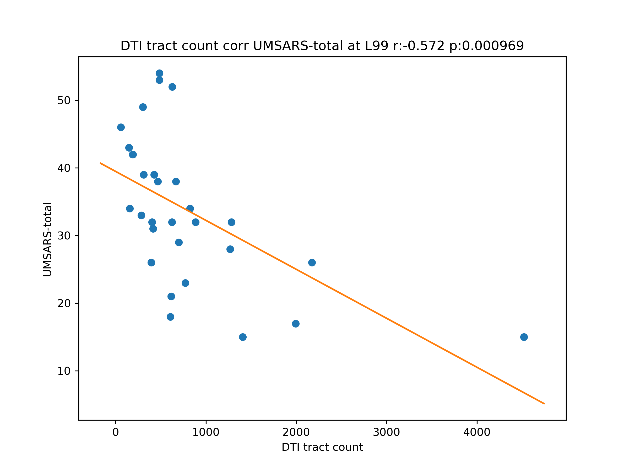


**Supplementary Figure 6**. Correlation result (left) and Q-Q plot (right) of DTI tract count at L99 (Cbe6.L). One obvious outlier (patient 21) was found.


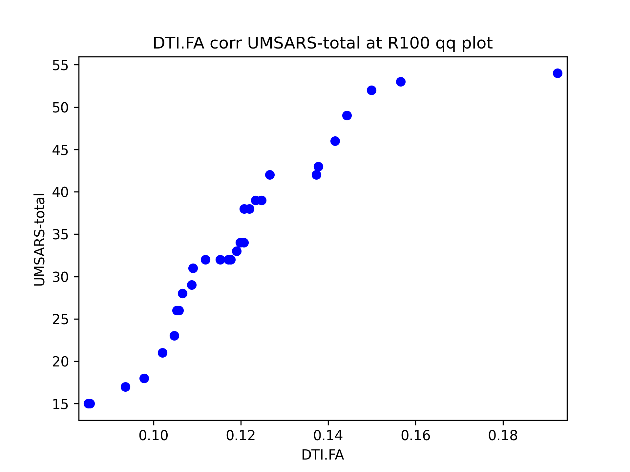

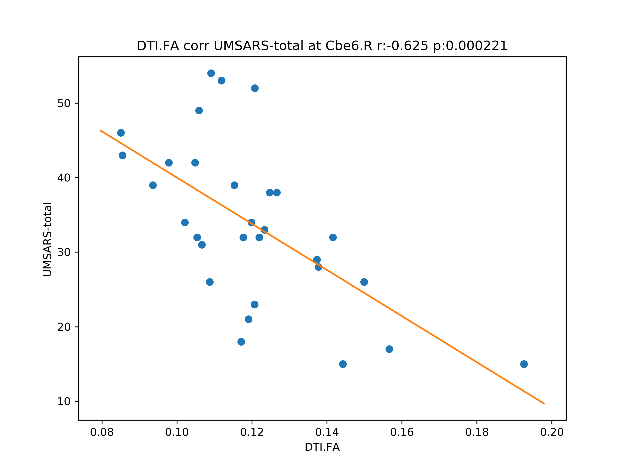


**Supplementary Figure 7**. Correlation result (left) and Q-Q plot (right) of DTI FA at R100 (Cbe6.R). One obvious outlier (patient 21) was found.


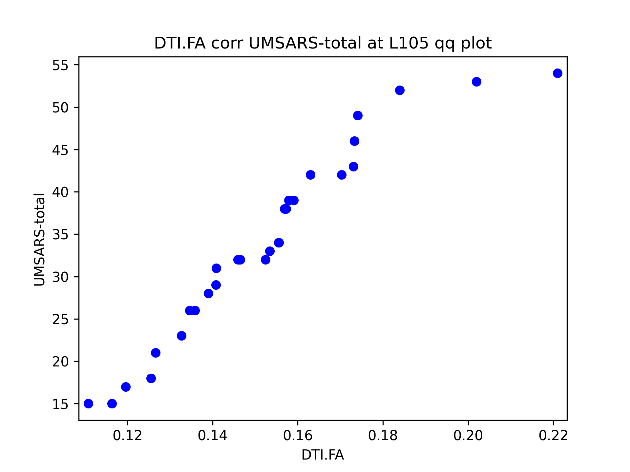

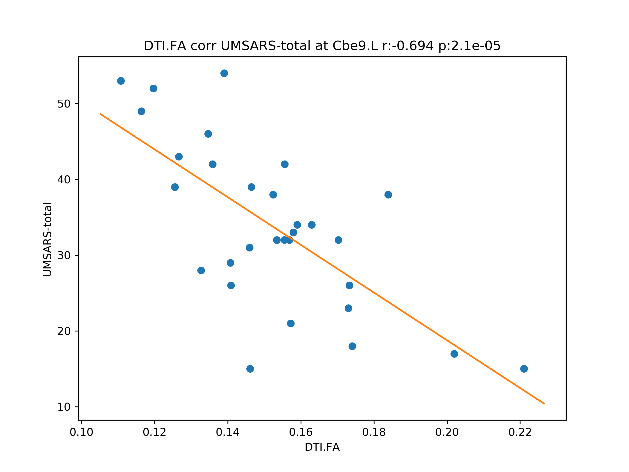


**Supplementary Figure 8**. Correlation result (left) and Q-Q plot (right) of DTI FA at L105 (Cbe9.L). One obvious outlier (patient 21) was found.


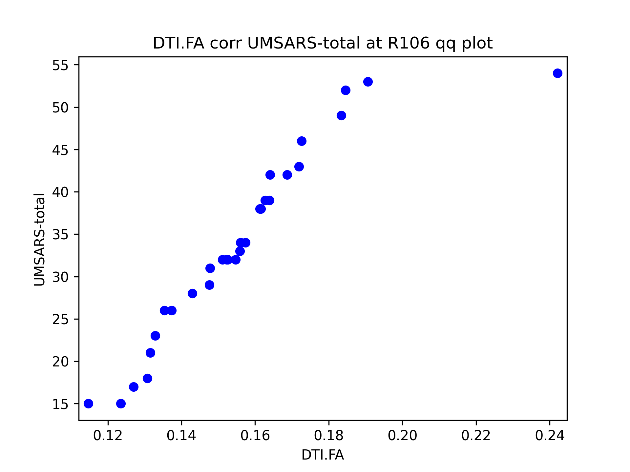

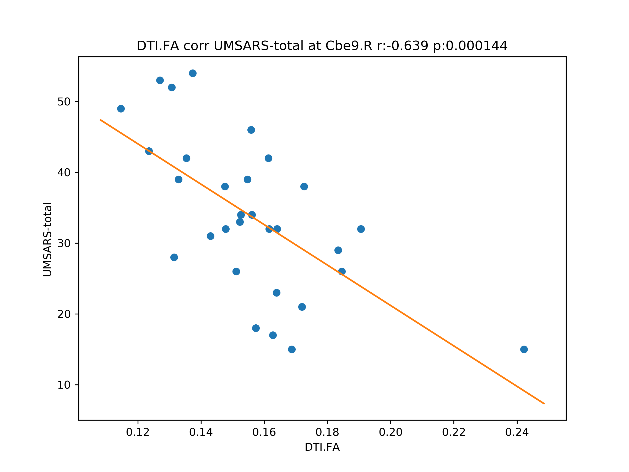


**Supplementary Figure 9**. Correlation result (left) and Q-Q plot (right) of DTI FA at R106 (Cbe9.R). One obvious outlier (patient 21) was found.

As can be seen from Supplementary Figure 3-9, patient 21 was an obvious outlier. We excluded patient 21 and repeated the correlation analysis with same procedure. Firstly, significant features were identified by comparing features of the remaining twenty-nine MSA-c patients and twenty-seven healthy controls using two-sample t-test with FDR correction. Features whose p-value < 0.001 were selected and correlated with UMSARS-total scores. We also analyzed the distribution of features and clinical measurements using Q-Q plots. The results were shown in Supplementary Figure 10-12.


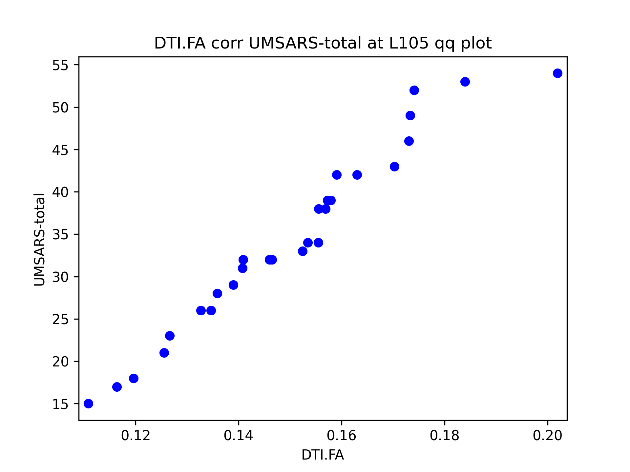

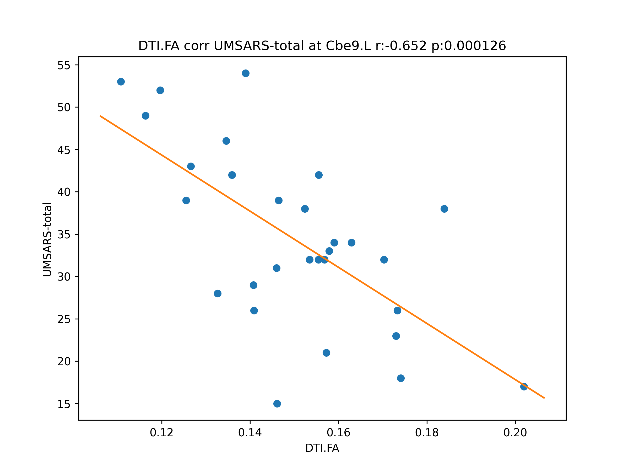


**Supplementary Figure 10**. Correlation result (left) and Q-Q plot (right) of DTI FA at L105 (Cbe9.L).


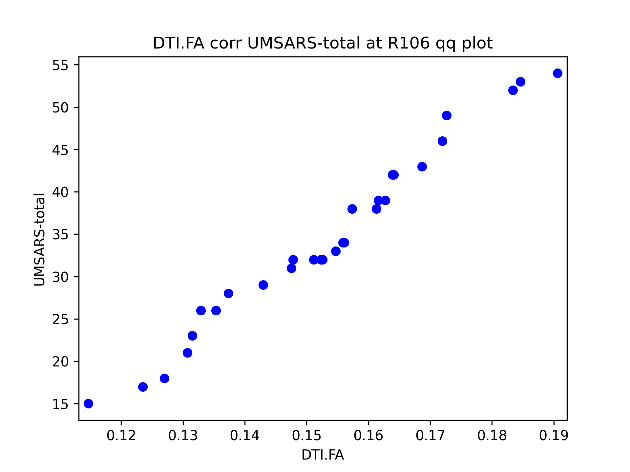

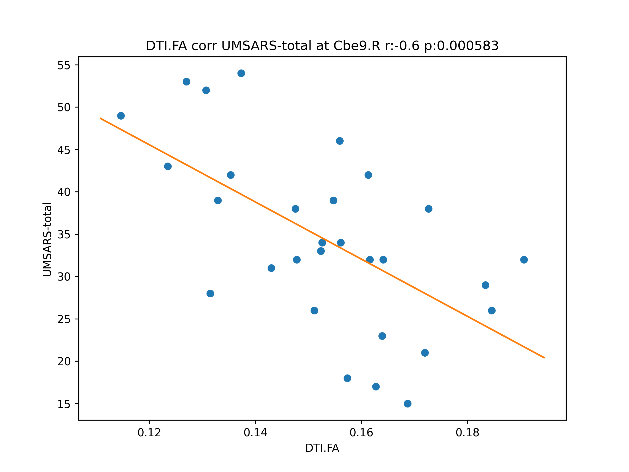


**Supplementary Figure 11**. Correlation result (left) and Q-Q plot (right) of DTI FA at R106 (Cbe9.R).


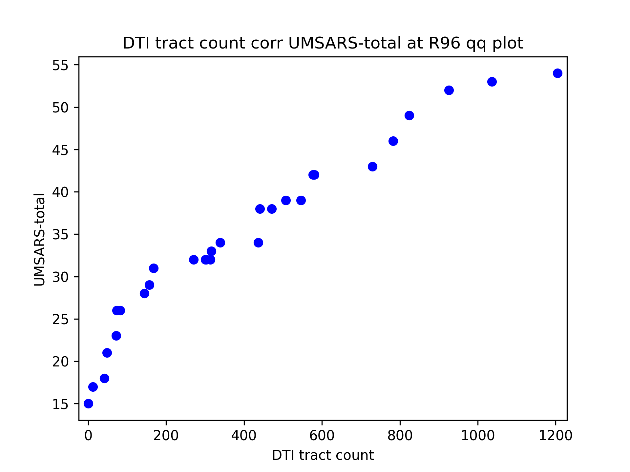

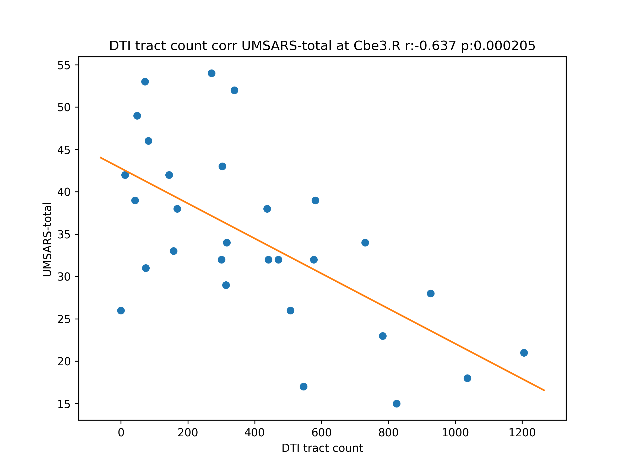


**Supplementary Figure 12**. Correlation result (left) and Q-Q plot (right) of DTI tract count at R96 (Cbe3.R).

After excluding patient 21, no obvious outlier was further identified. The statistical comparison, correlation analysis and classification were repeated for the remaining 29 MSA-c patients and 27 healthy controls. The results were reported in the main text and detailed statistics were reported in Supplementary Table 1-5.

## Supplementary Tables

**Supplementary Table 1**. Whole-brain statistical comparison result for grey matter volume (GMV) feature extracted from T1 images. Significant regions (p < 0.001, FDR corrected) were shown in the tick and region name column. Group means and standard deviations of the feature at each significant region for each group were reported in each row, along with the t-statistics and the corrected p-value after performing two-sample t-test.

The table was uploaded as a separate file (filename: SM Table 1 - T1.VBM.GMV CMSA 29 vs NC 27 0.001.csv).

**Supplementary Table 2**. Whole-brain statistical comparison result for white matter volume (WMV) feature extracted from T1 images. Significant regions (p < 0.001, FDR corrected) were shown in the tick and region name column. Group means and standard deviations of the feature at each significant region for each group were reported in each row, along with the t-statistics and the corrected p-value after performing two-sample t-test.

The table was uploaded as a separate file (filename: SM Table 2 - T1.VBM.WMV CMSA 29 vs NC 27 0.001.csv).

**Supplementary Table 3**. Whole-brain statistical comparison result for fractional anisotropy (FA) feature extracted from DTI images. Significant regions (p < 0.001, FDR corrected) were shown in the tick and region name column. Group means and standard deviations of the feature at each significant region for each group were reported in each row, along with the t-statistics and the corrected p-value after performing two-sample t-test.

The table was uploaded as a separate file (filename: SM Table 3 – DTI.FA CMSA 29 vs NC 27 0.001.csv).

**Supplementary Table 4**. Whole-brain statistical comparison result for mean diffusivity (MD) feature extracted from DTI images. Significant regions (p < 0.001, FDR corrected) were shown in the tick and region name column. Group means and standard deviations of the feature at each significant region for each group were reported in each row, along with the t-statistics and the corrected p-value after performing two-sample t-test.

The table was uploaded as a separate file (filename: SM Table 4 – DTI.MD CMSA 29 vs NC 27 0.001.csv).

**Supplementary Table 5**. Whole-brain statistical comparison result for fiber bundle count (DTI tract count) feature extracted from DTI images. Significant regions (p < 0.001, FDR corrected) were shown in the tick and region name column. Group means and standard deviations of the feature at each significant region for each group were reported in each row, along with the t-statistics and the corrected p-value after performing two-sample t-test.

The table was uploaded as a separate file (filename: SM Table 5 – DTI tract count CMSA 29 vs NC 27 0.001.csv).

**Supplementary Table 6**. Classification results for each brain region. The region was shown in the tick and region name column. Features used for classification were reported in the feature column. Feature_num shows the number of significant features used for classification for this region. Nested_cv_acc, nested_cv_sensitivity and nested_cv_specificity stands for the classification accuracy, sensitivity and specificity obtained using the nested leave-one-out cross-validation framework. The selected classification parameter was reported in the best_param column.

The table was uploaded as a separate file (filename: SM Table 6 - Classification regionwise linear result CMSA 29 vs NC 27.csv).
