# Supplementary figures and images for: Altered Brain Volume, Microstructure Metrics and Functional Connectivity Features in Multiple System Atrophy
Source: Front Aging Neurosci. 2022 May 19;14:799251. doi: 10.3389/fnagi.2022.799251 (PMC9162384; doi:10.3389/fnagi.2022.799251)

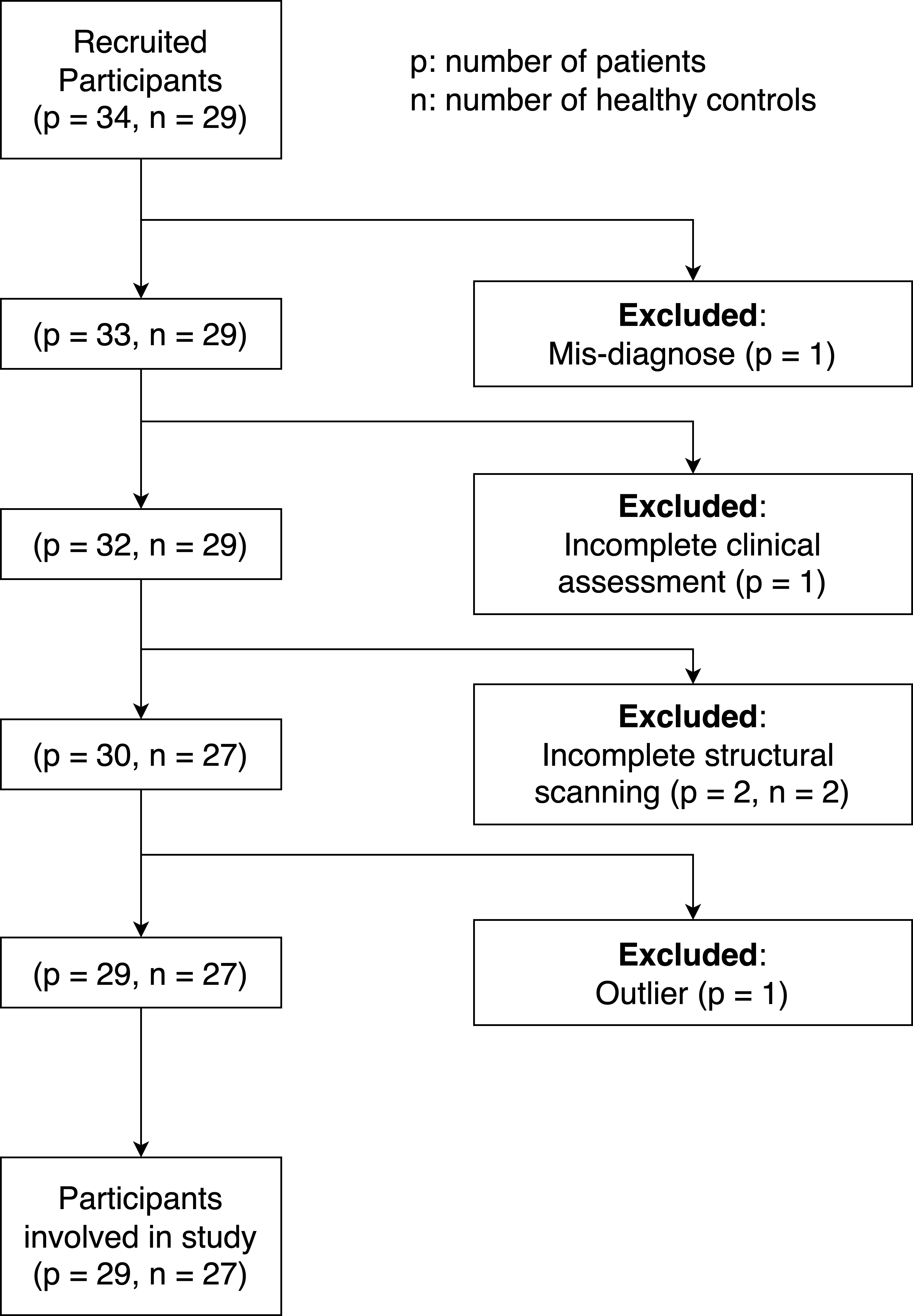

Supplement: Supplementary file 8 [file Image_1.PNG]

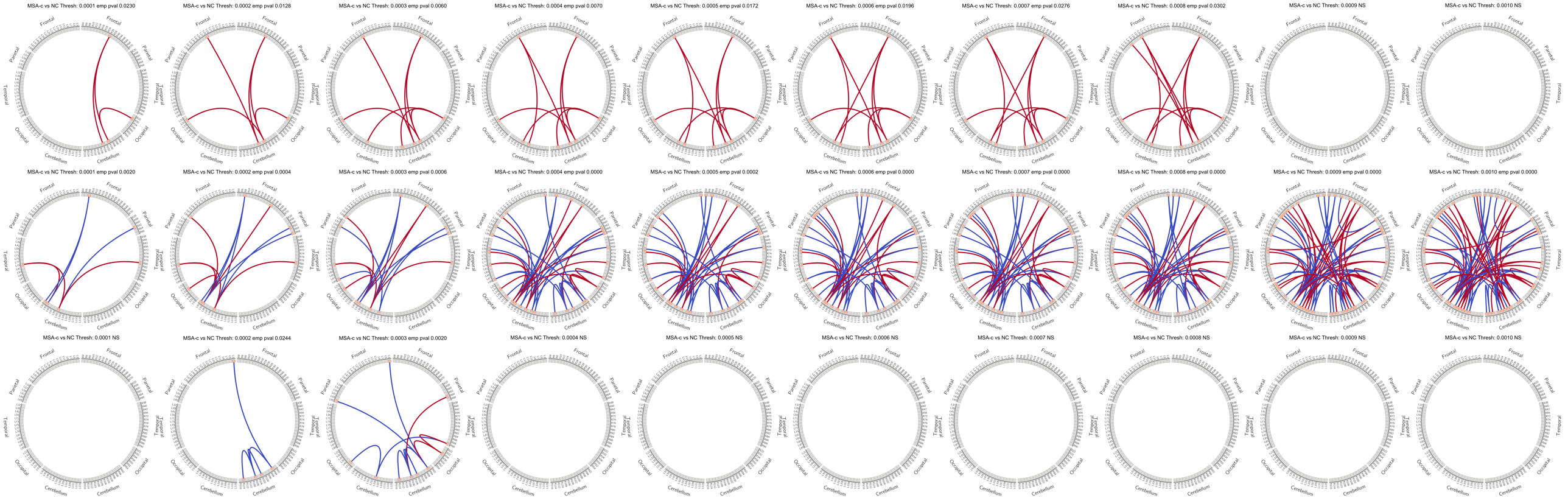

Supplement: Supplementary file 9 [file Image_2.PNG]
